# Seminoma in a Man with Russell-Silver Syndrome Presenting with Testicular Torsion

**DOI:** 10.1155/2016/6017636

**Published:** 2016-02-29

**Authors:** Satoshi Funada, Ryosuke Ikeuchi, Toru Yoshida, Takehiko Segawa

**Affiliations:** Department of Urology, Kyoto City Hospital, Kyoto, Japan

## Abstract

Russell-Silver syndrome (RSS) is a type of primordial dwarfism. Only one case of testicular cancer in RSS has been reported, the pathology of which was nonseminoma. Here, we report a case of seminoma in a 36-year-old man who was diagnosed with RSS at birth. The seminoma was diagnosed when the patient presented with testicular torsion. This is the first report of testicular seminoma in an RSS patient in the literature. We also discussed the correlation between seminoma and RSS.

## 1. Introduction

Russell-Silver syndrome (RSS) is a type of primordial dwarfism characterized by restricted intrauterine and postnatal growth, relative macrocephaly, triangular face, asymmetry, and feeding difficulties [1]. The average adult heights of RSS patients are as follows: male: 151.2 cm; female: 139.9 cm [2].

RSS is associated with hereditary abnormality and chromosomal anomaly. Of all RSS patients, 38.5% have hypomethylation of chromosome 11p15.5 [3], and 10% have maternal uniparental disomy for chromosome 7 [4].

Only one case of nonseminoma testicular cancer in an RSS patient has been reported [5]. Here, we report the first case of testicular seminoma in an RSS patient, which was diagnosed after presentation of testicular torsion, and examine the correlation between RSS and testicular cancer.

## 2. Case Presentation

**Figure 1:**
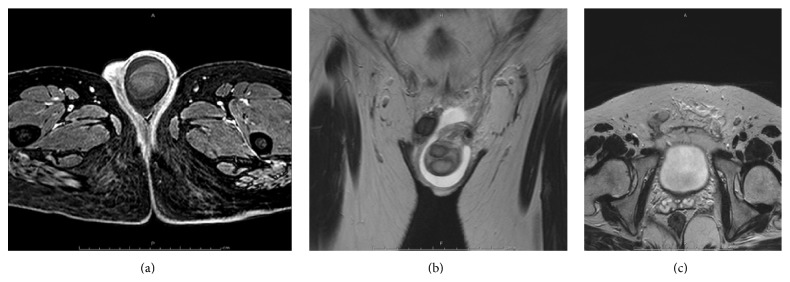
Contrast-enhanced magnetic resonance imaging. (a) No enhancement and inhomogeneous signal of the left testis. (b) Torsion of the left testis. (c) Cryptorchidism of the right testis.

A 36-year-old man presented with a painful left testis of 9 days' duration. He was diagnosed with RSS at birth. His height was 152.0 cm at the initial visit and he had a triangular face and asymmetrical limbs. Physical examination revealed mild swelling and pain in left testis. No inguinal lymphadenopathy was found on palpation. His serum germ cell tumor markers (lactate dehydrogenase, *β*-human chronic gonadotropin, and *α*-fetoprotein) were in normal range. Color Doppler sonography showed a hypoechoic and no flow in the left testis. Contrast MRI showed left testicular torsion with findings suggestive of tumor and right cryptorchidism (Figure 1). We saw no lymph node swelling on contrast computed tomography scans of the chest, abdomen, and pelvis. We suggested orchiectomy but the patient declined and returned home. However, he returned with fever and fatigue 5 days later and was admitted into hospital. He underwent a left high orchiectomy and a right orchiopexy.

**Figure 2:**
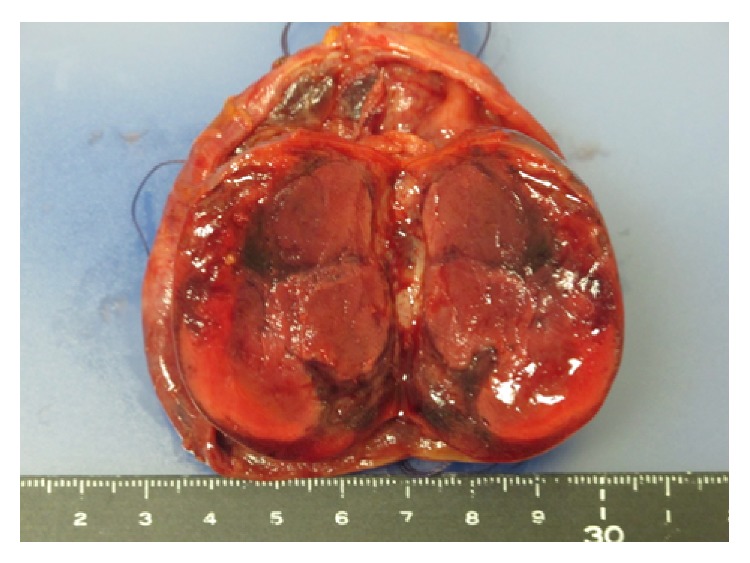
Gross appearance. Outside surface was blackened in spots; inside lumen was occupied by hematomas and necrotic tissue.

**Figure 3:**
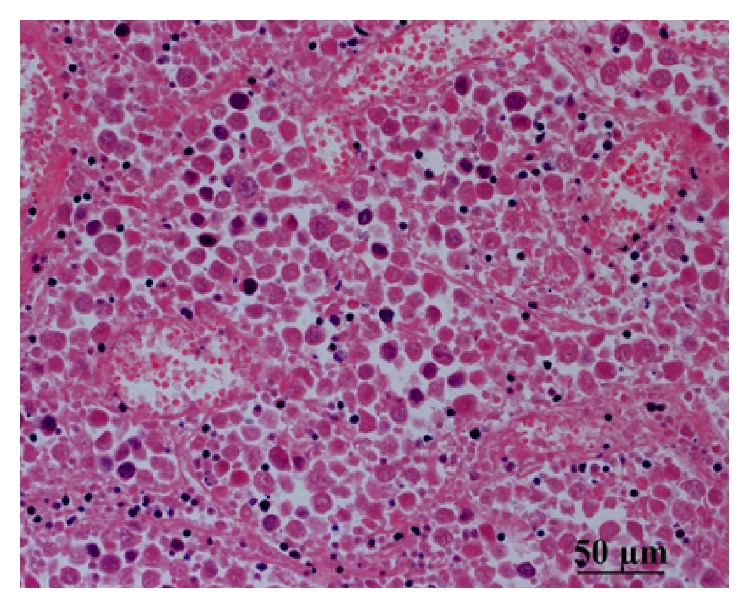
Left testis section (hematoxylin-eosin stain). Area was dominated by necrotic tissue, but some cells showed high nuclear/cytoplasmic ratios, suggestive of malignancy.

**Figure 4:**
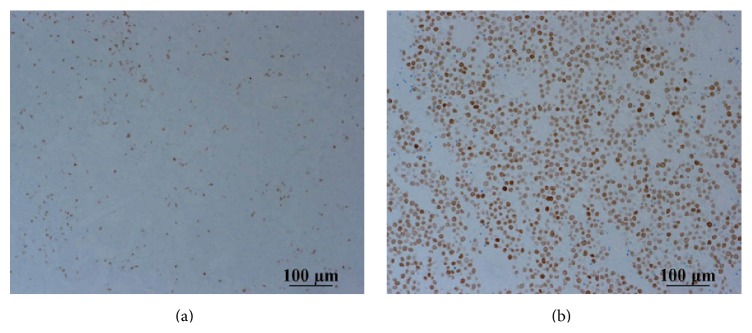
Left testis section. Immunostains for (a) CD79a (negative) and (b) SALL4 (positive).

In surgery, the left testis was found to be twisted at 270 degrees, with a blackened appearance. Although we untwisted the testis, its color did not return and we performed high orchiectomy. We also performed orchiopexy on the undescended right testis. Gross examination revealed that the outer surface was blackened in spots and the inside lumen was occupied with hematomas and necrotic tissue (Figure 2). Microscopic examination of the left testis showed that the area was dominated by necrotic tissue, but cells with high nucleic/cytoplasmic ratios were also seen, which suggested malignancy (Figure 3). Immunostains were positive for CD 3 and CD 20 but negative for c-KIT. To help distinguish seminoma from malignant lymphoma, we added immunostains for CD79a (result: negative) and SALL4 (positive; Figure 4).

Finally, we diagnosed testicular torsion of the seminoma. After surgery, the patient recovered well. He has been followed up for one year with no recurrence and no elevation of serum germ cell tumor markers.

## 3. Discussion

RSS is a type of primordial dwarfism characterized by restricted intrauterine growth, failure to thrive, and poor postnatal growth. Its estimated prevalence is one in 100,000 people [6]. The two reported causes of RSS are hypomethylation of chromosome 11p15.5 and maternal uniparental disomy for chromosome 7. RSS affects many physical features, usually manifesting with a small and triangular face, broad forehead, and genitourinary anomalies, such as cryptorchidism, hypospadias, and small testes and penis. To our knowledge, only one case of testicular cancer in an RSS patient has been reported in the English literature [5]. As it was a nonseminoma, the current case is the first report of seminoma in an RSS patient.

RSS has risk factors for seminoma development. First, cryptorchidism is a risk factor for testicular cancer. A meta-analysis of 21 studies associated undescended testes with a 4.8 overall relative risk of testicular germ cell tumors [7]. In our case, the right testis was undescended and the left was scrotal, but seminoma developed in the left side. The cohort study reported a 3.6 relative risk of testicular cancer in the normally descended testis of unilateral cryptorchid men [8], so the contralateral, normally descended testis in unilateral cryptorchid man has a high risk of testicular cancer.

Second, hypomethylation of chromosome 11p15.5, which occurs in 38.5% of RSS cases [3] and leads to hypomethylation of the* IGF2/H19* imprinting control region (ICR) [3], may be a risk factor for seminoma. Sievers et al. reported that most germ cell tumors showed low methylation at the* IGF2*/*H19* ICR [9]. In their study, 9 of 10 testicular seminomas had hypomethylated* IGF2/H19* ICRs (≤33% methylated). Thus, hypomethylated chromosome 11p15.5 may cause RSS and testicular germ cell tumors through an epigenetic mechanism.

Third, RSS itself is a risk factor for testicular dysgenesis syndrome (TDS), based on the hypothesis that environmental factors and genetic aberrations or polymorphisms decrease Leydig and Sertoli cell functions. This can possibly lead to four conditions: cryptorchidism, hypospadias, impaired spermatogenesis, and testicular cancer [10]. RSS fits in the TDS model because of genetic aberration (hypomethylation of chromosome 11p15.5 and maternal uniparental disomy for chromosome 7) and genitourinary anomalies (e.g., cryptorchidism, hypospadias, small testes, and penis), which implies that men with RSS are likely to develop testicular cancer. Lutke Holzik et al. reviewed earlier studies and reported correlations between hereditary disorders and constitutional chromosomal anomalies and testicular cancer [11]. They reported that RSS carries many risk factors for testicular cancer and discussed TDS with regard to hereditary disorders and constitutional chromosomal anomalies. RSS is very rare and molecular studies are needed to definitively correlate it with seminoma.

## 4. Conclusion

We here report a rare case of testicular cancer in a man with Russell-Silver syndrome; this cancer was detected with a presentation of testicular torsion, which makes it even rarer. To our knowledge, this is the first report of seminoma with RSS. Molecular studies are needed to clearly correlate RSS and seminoma.
